# Loop Recorder Detected High Rate of Atrial Fibrillation Recurrence after a Single Balloon- or Basket-Based Ablation of Paroxysmal Atrial Fibrillation: Results of the MACPAF Study

**DOI:** 10.3389/fcvm.2017.00004

**Published:** 2017-02-13

**Authors:** Alexander Schirdewan, Juliane Herm, Mattias Roser, Ulf Landmesser, Matthias Endres, Lydia Koch, Karl Georg Haeusler

**Affiliations:** ^1^Department of Cardiology, Sana Clinic Lichtenberg, Berlin, Germany; ^2^Department of Neurology, Charité – Universitätsmedizin Berlin, Berlin, Germany; ^3^Center for Stroke Research Berlin, Charité – Universitätsmedizin Berlin, Berlin, Germany; ^4^Department of Cardiology and Pneumology, Charité – Universitätsmedizin Berlin, Berlin, Germany; ^5^German Center for Neurodegenerative Diseases (DZNE), Partner Site, Berlin, Germany; ^6^German Center for Cardiovascular Diseases (DZHK), Partner Site, Berlin, Germany

**Keywords:** catheter ablation, pulmonary veins, atrial fibrillation, Arctic Front^®^, HD Mesh Ablator^®^, ECG monitoring

## Abstract

**Purpose:**

Pulmonary vein isolation (PVI) is an established approach to treat symptomatic non-permanent atrial fibrillation (AF). Detecting AF recurrence after PVI is important, if discontinuation of oral anticoagulation after ablation is considered.

**Methods:**

Patients with symptomatic paroxysmal AF were enrolled in the prospective randomized mesh ablator vs. cryoballoon pulmonary vein (PV) ablation of symptomatic paroxysmal AF study, comparing efficacy and safety of the HD Mesh Ablator^®^ (C.R. Bard, Lowell, MA, USA) and the Arctic Front^®^ (Medtronic, Minneapolis, MN, USA) catheter. Rhythm status post-PVI was closely monitored for 1 year using the implantable loop recorder (ILR) Reveal XT^®^ (Medtronic Minneapolis, MN, USA).

**Results:**

The study was terminated after the first interim analysis due to the inability of the HD Mesh Ablator^®^ to achieve the predefined primary study endpoint, an exit block of all PVs. After a 90-day blanking period, 23 (62.2%) out of 37 study patients (median 63.0 years; 41% females) had at least one episode of AF. AF recurrence was associated with AF episodes during the blanking period {hazard ratios (HR) 5.10 [95% confidence interval (CI) 1.21–21.4]; *p* = 0.038}, and a common left-sided PV ostium [HR 4.17 (95%CI 1.48–11.8); *p* = 0.039] but not with catheter type, age, gender, cardiovascular risk profile, or left atrial volume. There was a trend toward AF recurrence in patients without complete PVI of all PV (*p* = 0.095). Overall, 337 (59.4%) out of 566 ILR-detected episodes represented AF. Comparing patients with AF recurrence to those without, there was no difference in cognitive performance 6 months post-ablation.

**Conclusion:**

Using an ILR, in more than 60% of all patients with paroxysmal AF, a recurrence of AF was detected within 12 months after ablation. In patients with a common PV ostium, the first generation balloon-based catheter is obviously less effective.

**Clinical trials:**

http://Clinicaltrials.gov NCT01061931.

## Introduction

Atrial fibrillation (AF) increases stroke risk, impacts quality of life, and is associated with cognitive decline (1–3). Pulmonary vein isolation (PVI) is now an established therapeutic approach in patients with symptomatic paroxysmal or persistent AF (2). Besides reducing AF-related symptoms, ablation of pulmonary vein (PV) significantly improves quality of life (4).

The ability of PVI to assure continuous sinus rhythm (SR) on the long term is limited (5–7) but can be improved by repetitive ablation procedures. Reported “success rates” in AF patients strictly depend on AF type, patient characteristics, ablation techniques, and the extent of ECG recording after ablation (8–10). The implantable loop recorder (ILR) Reveal XT^®^ (Medtronic, Minneapolis, MN, USA) is a promising tool to monitor AF recurrence by detecting paroxysmal AF with a sensitivity of 96% and specificity of 85% (11), and it is more precise to detect AF recurrence after PVI compared to repetitive Holter monitoring (9). Assessment of AF recurrence is of paramount importance in AF patients with low or intermediate stroke risk if discontinuation of oral anticoagulation is considered (12). Moreover, AF recurrence is important to establish the (technical) efficacy and anatomical limitations of new (especially single shot) devices (13).

By implanting the ILR Reveal XT^®^ before PVI within the randomized “mesh ablator vs. cryoballoon pulmonary vein ablation of symptomatic paroxysmal atrial fibrillation” (MACPAF) study, we aimed to evaluate whether the cryoballoon Arctic Front^®^ (Medtronic, Minneapolis, MN, USA) or the radiofrequency-based HD Mesh Ablator^®^ catheter (C.R. Bard, Lowell, MA, USA) is able to achieve continuous SR after a single PVI procedure (14). As reported previously, the Arctic Front^®^ catheter proved to be superior by achieving a bidirectional block, entrance, and “exit block” (EB) of all PVs (15). We here report the ILR data during a 12-month follow-up as well as the impact of AF recurrence on cognitive performance.

## Materials and Methods

### Study Design and Study Population

The design of the MACPAF study was previously reported in detail (14, 15). In short, the study’s safety board terminated the study prematurely due to the inability of the HD Mesh Ablator^®^ to achieve the predefined primary study endpoint, an EB of all PVs. Of the intended 108 patients with symptomatic paroxysmal AF (with prior ineffective antiarrhythmic drug treatment, no previous PVI, no unstable structural heart disease), 37 patients were randomized for Arctic Front^®^ or HD Mesh Ablator^®^ catheter ablation. The balloon shaped catheter Arctic Front^®^ (Medtronic, Inc.) uses cryoenergy, whereas the basket shaped catheter device HD Mesh Ablator^®^ (C.R. Bard, Inc.) is based on unique pulsed radiofrequency delivery. In addition, the latter provides the possibility of circumferential mapping, leading to a reduced procedure duration as well as a reduced fluoroscopy time (16).

Study patients underwent PV ablation according to study criteria and were followed up 3, 6, 9, and 12 months afterward. Stable cardiac conditions were ensured before ablation by performing echocardiography, cardiovascular stress test, and (if indicated) coronary angiography. Using MRI or CT datasets, the LA-PVs were segmented to generate 3D LA-PV surface reconstructions. In MACPAF, PV ostial and antral regions were evaluated. A common left-sided ostium was defined as a common ostial circumference with left PVs branching >3 mm away from the common ostium (17).

A comprehensive cognitive testing was carried out before PVI and during the 6 months visit (18), including verbal and non-verbal learning (Rey–Osterrieth complex figure test and the Rey Auditory Verbal Learning Test), short term memory (forward digit-span task), attention and executive function (trail-making test A and B, Stroop test, category and letter fluency, digit-span backward), and reasoning (German Leistungsprüfsystem 50). The ILR Reveal XT^®^ (Medtronic Minneapolis, MN, USA) was implanted after enrollment in 33 (89%) out of 37 study patients to assess AF recurrence after ablation.

### Analysis of ECG Data

Atrial fibrillation was defined as absolute arrhythmia without distinguishable P waves lasting longer than 30 s. AF recurrence post-ablation was defined as one AF episode after a 90-day blanking period, verified by a board-certified cardiologist (LK, AS) blinded for patient reported symptoms during follow-up. Rhythm status was monitored using the ILR Reveal XT^®^ (*n* = 31) or repetitive Holter recordings for at least 24 h (range 1–7 days; *n* = 6), respectively. The subcutaneously inserted Reveal XT^®^ is able to store up to 49.5 min of automatically or patient activated ECG recording. Holter recording and Reveal XT^®^ data download were scheduled on the 3, 6, 9, and 12 months visit. The duration of available ECG recording was defined as time from first recording of the ILR to the scheduled read-out. AF burden was defined as total duration of all verified AF episodes during ECG recording.

### Statistical Analysis

For categorical traits, absolute and relative frequencies were computed. In the case of continuous or quasi-continuous variables with nearly symmetric distribution, the arithmetic mean, SD, minimal and maximal values, otherwise median, quartiles as well as minimal and maximal values were computed. Fisher’s exact test was used to compare proportions for dichotomous outcomes between independent groups or to test independency of two dichotomous traits within a population. The Mann–Whitney *U* test was applied to analyze not normally distributed variables. In order to analyze predictors of AF recurrence, AF-free survival time was computed. Univariate Cox proportional hazards analysis and log-rank test (Mantel Cox) were used for outcome analyses of AF-free survival. Hazard ratios (HR) and 95% confidence intervals (CI) are presented. The survival function was estimated by the Kaplan–Meier method (product-limit estimator). Due to the limited number of patients, all statistical tests have to be regarded as explorative. *p*-Values (significance level 0.05) are not adjusted for multiple testing.

## Results

### Patients’ Baseline Characteristics

The baseline data of the 37 study patients can be found in the Supplementary Material. In short, median age was 63.0 years (IQR 56.5–68.0), 40.5% were females and the median CHA_2_DS_2_-VASc score was 2.0 (IQR 1.0–3.0). All patients were available for the follow-up after 180 days; one (2.7%) patient with persisting SR was lost to follow-up afterward. No patient reported a clinically evident ischemic stroke or transient ischemic attack within 1 year post-PVI.

### Assessment of Rhythm Status and AF Recurrence during Follow-up

Due to local infection in two (5.4%) of 33 patients, these ILRs had to be explanted 19 and 35 days after implantation, respectively. Therefore, overall six (16.2%) study patients underwent serial Holter–ECG monitoring [median duration 5.0 days (IQR 1.0–5.0, range 1–7)] during the 1-year follow-up. After the blanking period of 90 days, 23 (62.2%) study patients had an ECG documented episode of AF within the next 270 days. In these 23 patients, the first documented AF episode occurred within 90 and 179 days in 17 (73.9%) patients, within 180 and 269 days in 5 (21.7%), and within 270 and 365 days in 1 patient (4.3%). AF recurrence was detected in 21 patients by using the ILR and in 2 patients by Holter monitoring. Within 90 days post-PVI, 22 (59.5%) of 37 study patients had at least 1 episode of AF. Five (22.7%) of these 22 study patients had documented AF only during the 90-day blanking period and were therefore considered to have continuous SR. Of all 23 study patients with recurrent AF, 13 (56.5%) underwent re-ablation within 1 year after first PVI.

### Predictors of AF Recurrence

**Figure 1:**
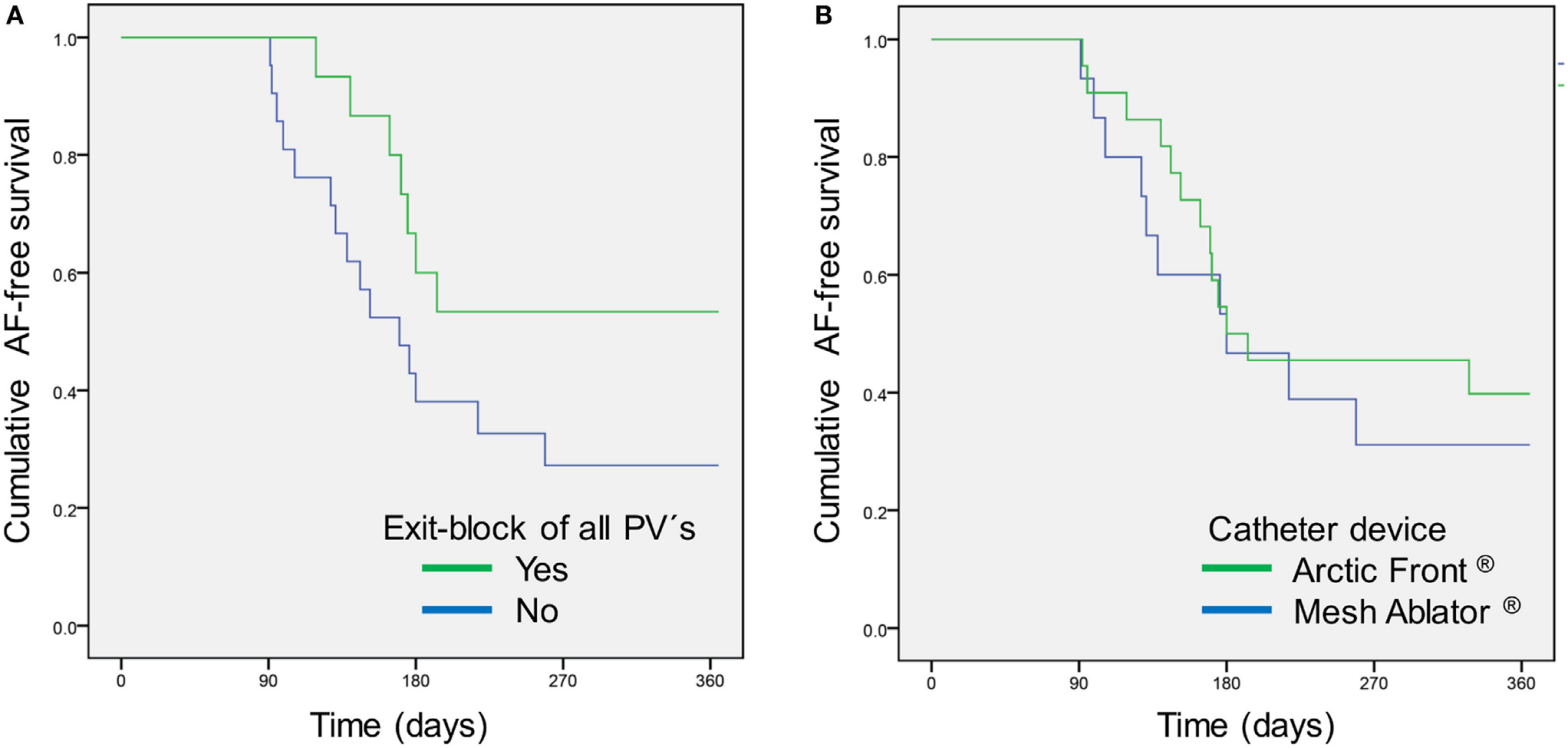
**Atrial fibrillation (AF)-free survival after pulmonary vein isolation (PVI) according to (A) achieved exit block of all pulmonary veins (PVs) during first PVI (*p* = 0.086) and (B) used catheter device (*p* = 0.547)**.

There was no difference regarding age, sex, or cardiovascular risk profile in patients with or without AF recurrence within 3–12 months post-PVI (Table 1). In addition, there was no impact of absence of any cardiovascular risk factor (“lone AF”) on AF recurrence. However, study patients with a common left-sided PV ostium were significantly more liable to suffer AF recurrence [HR 4.17 (95%CI 1.48–11.8); *p* = 0.039]. According to Kaplan–Meier analysis, AF-free survival is not related to the used catheter type (*p* = 0.547; Figure 1). There was a non-significant trend for patients with an EB of all PVs to be in continuous SR at 1-year follow-up [HR 0.46 (95%CI 0.19–1.14); *p* = 0.095] (Table 1). AF recurrence within the 90-day blanking period was significantly associated with AF recurrence during 90 and 365 days [Table 1; HR 5.10 (95%CI 1.21–21.4); *p* = 0.038].

**Table 1:** **Predictors of atrial fibrillation (AF) recurrence in patients with paroxysmal AF within 90 and 365 days after ablation**.

	AF recurrence		
	No (*n* = 14)	Yes (*n* = 23)	*p*
Age; years; median (IQR^a^)	63.5 (57.0–69.8)	63.0 (57.0–67.0)	0.544
Gender; female; % (*n*)	42.9 (6)	39.1 (9)	0.793
CHA_2_DS_2_-VASc; median (IQR)	2.0 (1.0–3.3)	2.0 (1.0–2.0)	0.191
Comorbidities; % (*n*)			
None (“lone” AF)	42.9 (6)	43.5 (10)	0.544
Heart failure	0 (0)	4.3 (1)	0.904
Arterial hypertension	57.1 (8)	52.2 (12)	0.320
Diabetes mellitus	14.3 (2)	13.0 (3)	0.561
Previous stroke	14.3 (2)	4.3 (1)	0.318
Coronary artery disease	28.6 (4)	17.4 (4)	0.381
LV-EF^b^; %; median (IQR)	67.5 (64.8–70.0)	65.0 (60.0–70.0)	0.182
Left atrial volume; ml; median (IQR)	77.8 (61.9–101.5)	96.7 (70.4–108.3)	0.304
Creatinine; μmol/l; median (IQR)	84.4 (72.7–96.4)	78.7 (69.8–90.2)	0.884
Pulmonary vein (PV) anatomy; % (*n*)			
Common left-sided ostium	0 (0)	21.7 (5)	**0.007**
Accessory right PV	7.1 (1)	13.0 (3)	0.801
Use of HD Mesh Ablator^®^; % (*n*)	35.7 (5)	43.5 (10)	0.549
Use of Arctic Front^®^; % (*n*)	65.3 (9)	56.5 (13)	0.549
Exit block of all PVs; % (*n*)	57.1 (8)	30.4 (7)	0.095
Isolated PVs/patient; *n*; median (IQR)	4.0 (1.5–4.0)	2.0 (0.0–4.0)	0.062
Procedure duration; min; median (IQR)	209.0 (188.3–275.0)	206.0 (192.0–230.0)	0.936
AF recurrence within 90 days post-pulmonary vein isolation	5 (22.7)	17 (77.3)	**0.038**

*The bold text indicates statistical significance*.

*^a^Interquartile range*.

*^b^Left ventricular ejection fraction*.

### Reliability of AF Detection According to the ILR Reveal XT^®^

**Figure 2:**
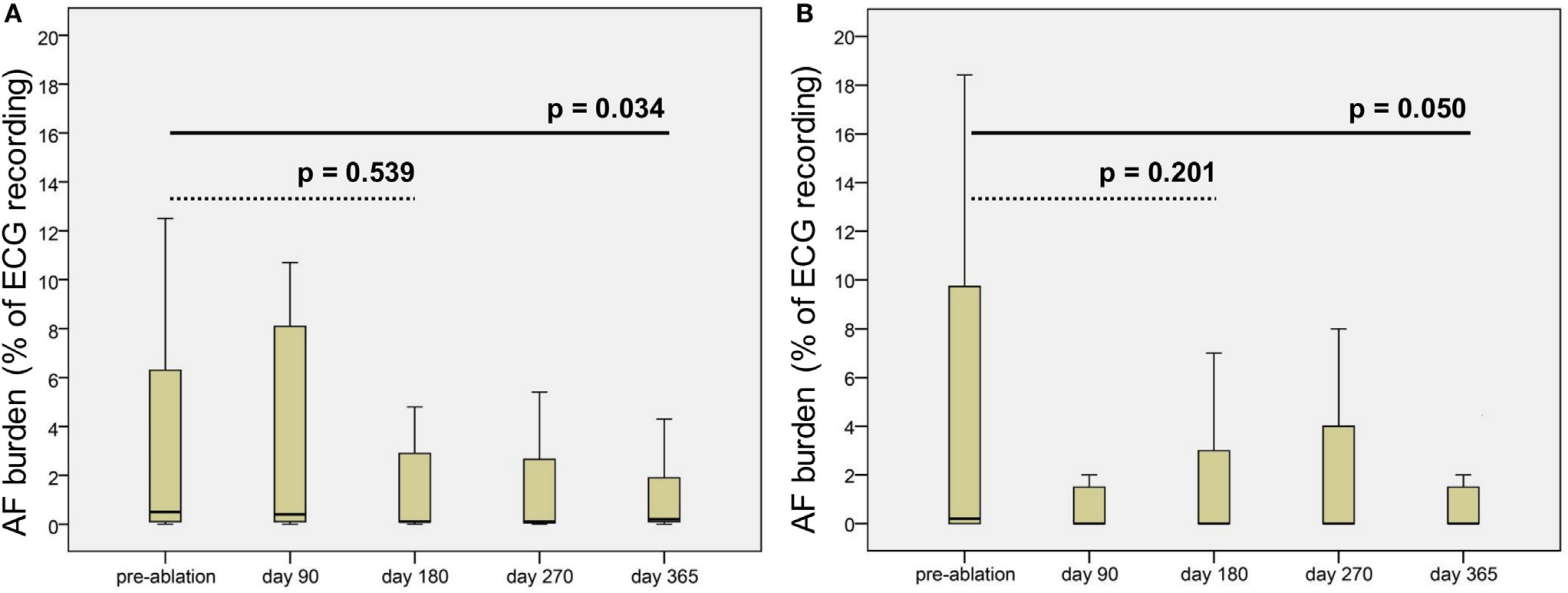
**Boxplots of (A) automatically detected atrial fibrillation (AF) burden (%) by the implantable loop recorder and (B) computed AF burden of verified AF episodes pre-ablation and during follow-up**. *p* values computed by the Wilcoxon signed-rank test (dotted line pre-ablation vs. day 180 post-ablation and solid line pre-ablation vs. day 365 post-ablation).

The ILR Reveal XT^®^ stored a median of 15.0 (IQR 6.0–63.0; range 1–1,040) episodes of suspected AF per patient and follow-up visit. Median duration of assessable ECG monitoring was 48.1 days (IQR 14.9–84.2) per follow-up. According to the algorithm of the ILR (11), a median AF burden of 0.55% (IQR 0.1–4.8) was present in the automated analysis. Overall, 337 (59.4%) of 566 episodes recorded by the ILR were verified as AF by the involved cardiologists. Frequent premature beats, under and oversensing of R waves, and a high electrical noise factor caused misclassification in the remaining episodes. A median of 9.0 (IQR 4.0–10.0) AF episodes per patient and follow-up was validated. Median duration of assessable Holter monitoring was 48.1 days (IQR 14.9–84.2) per follow-up. Median burden of verified AF in ILR patients was 0.21% (IQR 0–10.0) pre-ablation, 0.0% (IQR 0–4.5) on day 180, and 0.0% (IQR 0–2.0) on day 365, while median burden of automatically detected AF episodes by the ILR was 0.50% (IQR 0.1–9.1) pre-ablation, 0.1% (IQR 0.1–3.8) on day 180, and 0.2% (IQR 0.1–2.1) on day 365 (Figure 2). The Wilcoxon signed-rank test indicated a reduction of the verified AF burden (*p* = 0.05) as well as the automatically detected AF burden after 365 days (*p* = 0.034) compared to the pre-ablation period (Figure 2).

### Symptomatic AF and Medication during Follow-up

Atrial fibrillation-related symptoms like palpitations and dizziness were reported by 13 (76.5%) of 17 patients with AF recurrence on day 180. In addition, 4 (20%) of 20 patients without AF recurrence reported similar symptoms on day 180. On day 365, such symptoms were reported in 13 (56.5%) of 23 patients with AF recurrence and in 3 (23.1%) of 13 patients without (*p* = 0.083). In patients with “symptomatic” AF recurrence, recorded AF burden was significantly higher compared to those with “asymptomatic” AF recurrence [median 0.2% (IQR 0.08–0.48) vs. median 2.5% (IQR 0.55–5.28); *p* = 0.010].

On 1-year follow-up, 11 (31.4%) of all 36 study patients received at least one antiarrhythmic drug (2.9% amiodarone, 11.4% dronedarone, 17.1% other). Only one (9.1%) out of 13 patients without AF recurrence still received antiarrhythmic drugs. In addition, 27 (77.1%) of 36 patients were on oral anticoagulation, while 7 (19.4%) received an antiplatelet agent.

### Impact of AF Recurrence on Cognitive Performance

Neuropsychological testing was carried out in 36 (97%) of 37 patients during the 6-month follow-up visit. No patient complained about cognitive impairment during follow-up. Comparing patients with AF recurrence between 90 and 180 days to those without revealed no significant differences regarding all tested domains (Table 2).

**Table 2:** **Neuropsychological assessment pre-ablation and 6 months post-ablation according to atrial fibrillation (AF) recurrence**.

	AF recurrence >90 days post-ablation				
	No (*n* = 20/20)		Yes (*n* = 16/17)		
	
	Pre-LACA	Day 180	Pre-LACA	Day 180	p*
**Attention and executive functions**					
Trail-making test A; *s*	36 (27–47)	38 (29–56)	39 (26–43)	30 (25–43)	0.262
Trail-making test B; *s*	89 (77–129)	84 (67–120)	74 (65–102)	67 (60–108)	0.149
Color–word-interference test (Stroop); *s*	153 (141–182)	136 (125–172)	156 (135–160)	124 (105–142)	0.077
Category fluency; *n*	24 (18–30)	26 (21–31)	26 (21–31)	28 (24–30)	0.888
Letter fluency; *n*	17 (11–19)	14 (9–22)	17 (11–19)	17 (15–20)	0.286
Digit-span backward; points	8 (7–9)	8 (6–9)	8 (7–10)	9 (8–11)	0.189
**Short-term memory**					
Digit-span forward; points	6 (5–7)	6 (5–8)	7 (6–10)	9 (6–11)	0.369
**Learning (verbal and non-verbal)**					
RAVLT^a^; *n*	1 (−1 to 3)	9 (8–11)	0 (−3 to 5)	10 (7–11)	0.832
ROC figure^b^; points	23 (20–27)	26 (24–28)	30 (25–32)	27 (26–32)	0.779
**Reasoning**					
LPS 50^c^; points	21 (16–24)	21 (17–25)	23 (20–26)	3 (0–4)	0.236

*p values based on exact Mann–Whitney test.

*^a^Rey Auditory Verbal Learning Test (German Version); delayed recall (A7)*.

*^b^Rey–Osterrieth complex figure; immediate recall*.

*^c^Subtest 3 from the German Leistungsprüfsystem (LPS)*.

*Values are expressed as median (IQR)*.

## Discussion

Based on the here reported long-term results of the MACPAF study, the following main findings can be drawn from the study: using an ILR, 62% of all study patients with paroxysmal AF had recurrent AF within 90 days and 1-year post-PVI. AF recurrence during the 90-day blanking period was associated with a fivefold higher risk of AF recurrence. Patients with a common left-sided PV ostium had a fourfold higher risk of AF recurrence after a single ablation procedure, indicating that a first generation balloon- or basket-based catheter is less effective in these patients. There was no apparent impact of AF recurrence on cognitive performance 6 months post-PVI as determined by testing attention, executive functions, short term memory, verbal and non-verbal learning as well as reasoning.

In recent years, multiple innovative technical solutions for left atrial catheter ablation of symptomatic AF have been developed such as the balloon-based Arctic Front^®^ catheter (using cryoenergy) or the basket-based HD Mesh Ablator^®^ catheter (using pulsed radiofrequency), providing the opportunity to induce circumferential scars around the PV ostia (antral aspect) (19). However, the missing ability of the investigated first generation devices to do focal ablation is of practical importance regarding efficacy. The limited device efficacy and the relatively small patient cohort may explain the missing statistical significance regarding AF-free survival which was observed in other studies (20). A statistical trend toward less AF recurrences in patients with EB of all PVs further supports that transmural permanent PVI is an important factor of long-term success.

While these first generation devices obviously did not fit to all distinct PV anatomies, a substantial proportion of patients with paroxysmal AF suffered from mostly asymptomatic AF recurrences after PVI (21–23), and MACPAF patients with a common left-sided PV ostium were four times more liable to suffer AF recurrence [HR 4.17 (95%CI 1.48–11.8)]. Our results confirm an early prediction by Ahmed et al. that—based on the analysis of 3D surface reconstructions of LA-PV anatomy from MRI datasets in 101 patients—balloon or basket catheter-based ablation of the common PV antra would be problematic (17). This anatomical variant was found in 14% of all MACPAF patients compared to a prevalence of 2–7% in other ablation cohorts. This probably explains the missing association with AF recurrence in studies using similar ablation catheters (16, 24).

Analyzing the high rate of AF recurrence, the achieved results by the first generation cryoballoon are comparable to radiofrequency systems. The improved engineering of the second-generation cryoballoon leads to substantially higher efficacy also in patients with anatomical PV variants (25, 26). On the other hand, the inefficacy of the basket-based Mesh catheter raised serious concerns regarding the underlying technical concept.

Confirming previous studies (27, 28), patients with AF recurrence during the (well established) 90-day blanking period had a fivefold higher risk for later AF recurrence. However, about 23% of all study patients had documented AF only during the 90-day blanking period and were considered to have persisting SR after PVI. This finding is not in line with a previous study on 35 patients with paroxysmal AF (29), reporting AF recurrence in all 13 patients with AF during the blanking period. In conclusion, we do not think that the so-called blanking period loses its intrinsic meaning. We suggest that (even asymptomatic) patients with documented AF recurrence within 90 days after ablation should undergo intense ECG monitoring if the CHA_2_DS_2_-VASc score is ≥2 and long-term oral anticoagulation—despite current guideline recommendations (2, 30, 31)—is not intended.

The method and intensity of rhythm surveillance has become a major topic of post-ablational care. As an example, the randomized RAAFT 2 trial demonstrated a recurrence rate of AF/atrial flutter/atrial tachycardia of 24% by using standard Holter ECGs and a recurrence rate of 47% in the 2-year follow-up by using an additional transtelephonic monitoring (32). Available prospective ILR-studies on AF recurrence after PVI are mostly based on single-center experience, report on a limited number of AF patients, and are heterogeneous regarding AF type, definition of AF recurrence, ablation technique as well as duration of follow-up (9, 10, 29, 32, 33). Subsequently, there are few comparable data to the here reported AF recurrence rate of 62% within 1 year after a single balloon-based PVI of symptomatic paroxysmal AF. Using a similar definition of AF recurrence, Pedrote et al. reported an AF recurrence in 43% of 35 patients with paroxysmal AF within 1 year after a single PVI using radiofrequency (29). A recent propensity score-matched comparison of radiofrequency or cryoballoon ablation in 142 patients with paroxysmal AF reported similar long-term recurrence rates of 44 or 52% after a single ablation procedure, respectively (34). By using a threshold of “percentage of time spent in AF <0.5%” for considering a patient “free” of AF, a recurrence rate of 32% was reported for similar cohorts of patients with paroxysmal AF undergoing a single ablation procedure using radiofrequency (10, 33). In this context, the feasibility of discontinuation of oral anticoagulation within months post-PVI in AF patients with at least moderate stroke risk remains disputable.

While the use of ILRs is feasible (9, 10, 21, 29, 33) and more effective compared to repetitive Holter monitoring (9), automated recording of AF by the ILR Reveal XT^®^ is prone to false positive results (9). Our data revealed that about 60% of the ILR-detected “AF”-episodes could be verified by board-certified cardiologists. As similarly reported by Kapa et al. (9), main causes of false-positive AF detection by the ILR were frequent atrial and ventricular extrasystoles, undersensing of R waves, oversensing of R and T waves, and a high electrical noise factor. ILR misclassification (41% false-positive) was comparable to Kapa et al. [54% false-positive (9)] and Eitel et al. [38% false-positive (35)] but higher compared to Schmidt et al. [26% false-positive (36)]. While adjusting of the diagnostic algorithm has previously proven to improve specificity (35), further improvements are needed. Despite of being technically outdated by the Reveal LINQ^®^, socioeconomic reasons are likely to lead to an ongoing use of the Reveal XT^®^. Next to establishing continuous SR, control of AF-related symptoms is the main goal of ablation therapy. In our cohort, 43% of patients with AF recurrence reported to be free of AF-related symptoms after 12 months. AF burden was significantly reduced in those patients after a single ablation procedure, confirming the concept of single shot devices in principle. This confirms previous studies reporting a reduced rate of symptomatic AF and an improved quality of life even in ablated patients without continuous SR (4, 23).

In addition to AF-related symptoms, cognitive decline has been reported in AF patients (3, 37, 38) as well as in AF patients post-ablation (39). Whether continuous SR post-AF ablation might have a positive impact on cognitive function has not yet been reported. Our results demonstrate that rhythm status had no significant impact on cognitive function over a period of 180 days after ablation. However, large prospective multicenter studies are warranted to clarify this matter.

The study has several strengths but also limitations. While the study design is unique, the premature termination of the study limits the significance of the reported results. Subsequently, we cannot definitively prove that there was no impact of the absence of any cardiovascular risk factor (“lone AF”) on AF recurrence. In addition, PVI-related parameters such as procedure duration or the used catheter type in this randomized study had no impact on AF recurrence, despite of the fact that the HD Mesh Ablator^®^ catheter was inferior to the Arctic Front^®^ catheter in achieving an EB of all PVs (15). Furthermore, MACPAF results cannot be generalized to patients with persistent AF or distinct catheter types. In addition, the vast majority but not all patients were monitored by an ILR, and one patient was lost to follow-up after the 6 months visit.

However, the MACPAF results deliver relevant insights into important practical aspects of ILR-based follow-up performance. Moreover, our findings indicate that first generation balloon-based devices should not be used in patients with a common PV ostium.

## Conclusion

Using an ILR in 62% of all study patients with paroxysmal AF a recurrence of AF was detected within 90 days and 12 months after a single balloon-based ablation procedure. While AF burden was diminished after a single ablation procedure in general, about one third of patients with AF recurrence still suffered from symptomatic AF. Detected AF recurrence during the blanking period of 90 days post-ablation renders later AF recurrence fivefold more likely. A common left-sided PV ostium was associated with a fourfold higher risk of AF recurrence, indicating that a single balloon-based ablation procedure is less effective in these patients. Despite the fact that AF recurrence post-ablation had no significant impact on cognitive function 6 months after PVI, our study clearly demonstrates that intensive screening for AF recurrence after ablation is of major importance.

## Ethics Statement

The study has been approved by the local Ethics Committee (EA4/087/08). All the study participants gave written informed consent for study participation and scientific use of data.

## Author Contributions

AS has made substantial contributions to conception and design, analysis and interpretation of data, and drafted the manuscript. JH has made substantial contributions to analysis and interpretation of data, and drafted the manuscript. LK has made substantial contributions to conception and design, analysis of data, and revised the manuscript critically for important intellectual content. MR, UL, and ME revised the manuscript critically for important intellectual content. KGH has made substantial contributions to conception and design, analysis and interpretation of data, and drafted the manuscript.

## Conflict of Interest Statement

AS reports lecture fees and prior study grants by Medtronic, C.R. Bard, and Biotronik. ME reports funding from Bayer and fees paid to the Charité from Amgen, Bayer Healthcare, BI, BMS, EVER, GSK, Pfizer, Novartis, and Sanofi. LK reports lecture fees by Medtronic and a Biotronik-sponsored fellowship. UL received consultant or lecture honoraria from St. Jude. KGH reports lecture fees and study grants by Bayer Healthcare and Sanofi as well as lecture fees from Pfizer and Bristol-Myers Squibb. KGH received advisory board fees from Bayer Healthcare, Pfizer, Medtronic, and Edwards Lifesciences. The remaining authors declare no conflict of interest. The reviewer CT and handling Editor declared their shared affiliation, and the handling Editor states that the process nevertheless met the standards of a fair and objective review.

## Abbreviations

AF, atrial fibrillation; EF, ejection fraction; LA, left atrial; LV, left ventricular; MACPAF study, mesh ablator vs. cryoballoon pulmonary vein ablation of symptomatic paroxysmal atrial fibrillation study; PV(s), pulmonary vein(s); SR, sinus rhythm.
