# Supplementary material for: Loop Recorder Detected High Rate of Atrial Fibrillation Recurrence after a Single Balloon- or Basket-Based Ablation of Paroxysmal Atrial Fibrillation: Results of the MACPAF Study
Source: Front Cardiovasc Med. 2017 Feb 13;4:4. doi: 10.3389/fcvm.2017.00004 (PMC5303896; doi:10.3389/fcvm.2017.00004)
Supplement: Supplementary file 1 [file table_1.docx]

**ONLINE SUPPLEMENT**

Baseline characteristics of the 37 MACPAF patients.

| Age; years; median (IQR) | 63.0 (56.5-68.0) |
| --- | --- |
| Gender; female; n (%) | 40.5 (15) |
| CHA_2_DS_2_-VASc score; median (IQR) | 2.0 (1.0-2.0) |
| Comorbidities; n (%) |  |
| None (“lone” AF) | 43.2 (16) |
| Heart failure | 4.3 (1) |
| Arterial hypertension | 54.1 (20) |
| Diabetes mellitus | 13.5 (5) |
| Previous stroke | 8.1 (3) |
| Coronary artery disease | 21.6 (8) |
